# Localized Choriocapillaris Flow Deficits at Perforating Vessel Sites in Myopic Eyes

**DOI:** 10.1167/iovs.67.1.43

**Published:** 2026-01-20

**Authors:** Alberto Quarta, Alessandro Feo, Francesco Romano, Enrico Borrelli, Federico Corvi, Umberto Palumbo, Chiara Olivieri, Giulia Corradetti, Rouzbeh Abbasgholizadeh, Mai Alhelaly, Shinichiro Chujo, Marko Popovic, Ceren Soylu, Raiyna Rattu, Muneeswar G. Nittala, Michele Reibaldi, Giovanni Staurenghi, Srinivas R. Sadda

**Affiliations:** 1 Doheny Eye Institute, Pasadena, California, United States; 2 Department of Ophthalmology, David Geffen School of Medicine, University of California, Los Angeles, California, United States; 3 Department of Neurosciences, Imaging and Clinical Sciences, University “G. d'Annunzio” Chieti-Pescara, Chieti, Italy; 4 Eye Clinic, Department of Biomedical and Clinical Science “Luigi Sacco”, Sacco Hospital, University of Milan, Milan, Italy; 5 Department of Surgical Sciences, University of Turin, Turin, Italy; 6 Department of Ophthalmology, “City of Health and Science” Hospital, Turin, Italy; 7 Ophthalmology Department, Tanta University, Egypt; 8 Department of Ophthalmology, Mie University Graduate School of Medicine, Tsu City, Mie, Japan; 9 Department of Ophthalmology and Vision Sciences, University of Toronto, Toronto, Canada

**Keywords:** perforating vessels, choriocapillaris flow deficits, myopia

## Abstract

**Purpose:**

To investigate the association between perforating scleral vessels (PSVs) and choriocapillaris (CC) flow deficits (CCFDs) in otherwise healthy myopic eyes, and to identify predictors of localized flow deficits at PSV entry sites.

**Methods:**

PSVs were localized using a combined en face and B-scan approach from swept-source OCTA volumes. CCFD% were quantified from signal-compensated 16-µm en face slabs using the Phansalkar method. Outcome measures included flow deficits at PSV sites (FDPSV), within 250 µm (FD250), and total CCFD. Mixed-effects regression was used to assess associations with demographic, refractive, and anatomic factors.

**Results:**

Fifty-two eyes from 31 healthy myopic (≤−0.5D) participants were included in the study. A total of 119 PSVs were identified. Correlation analyses demonstrated significant associations between FDPSV and age, axial length (AXL), and global CCFD (*P* < 0.05). In univariable analysis, older age and higher global CCFD (*P* < 0.001) were associated with increased FDPSV. In multivariable analysis, age (*β* = 0.23, *P* = 0.045), global CCFD (*β* = 1.02, *P* < 0.001), and PSV area (*β* = 16.5, *P* = 0.009) were significant predictors of FDPSV. AXL was inversely associated with FDPSV (*β* = −2.37, *P* = 0.010).

**Conclusions:**

Localized CCFDs were consistently observed at PSV sites in myopic eyes. Although higher age, global CCFD, and PSV area were associated with greater localized deficits, longer AXL was associated with lower CCFDs, possibly reflecting scleral remodeling or altered PSV orientation in more significant myopia. These findings highlight the relevance of PSVs as early modulators of CC flow before the development of pathologic myopia.

High myopia is a leading cause of irreversible vision loss worldwide. It is particularly prevalent in East Asian populations, and its incidence is projected to rise dramatically over the coming decades.^1^^–^^3^ Elongated axial length (AXL) induces structural and vascular changes that predispose to complications such as lacquer cracks (LC), myopic choroidal neovascularization (mCNV), and chorioretinal atrophy.^4^

Among the anatomical features increasingly recognized in myopic eyes are perforating scleral vessels (PSVs), which are often visualized with en-face swept-source optical coherence tomography (OCT) in combination with cross-sectional B-scans.^5^^–^^8^ PSVs are presumed to be short posterior ciliary arteries (SPCAs), the primary vascular conduits supplying the posterior choroid.^5^^,^^9^^,^^10^ Multiple studies have linked PSVs to sites of subsequent pathological change. Querques et al.^10^ reported that the majority of LC occur directly overlying PSVs, and Ishida et al.^6^ documented frequent proximity of PSVs to active mCNV lesions, suggesting that these vessels may influence these myopic complications. The mechanism underlying these associations remains incompletely understood. One hypothesis is that the SPCAs may exert focal mechanical pressure or alter tissue compliance at their exit points, contributing to microstructural stress in the overlying choroid and retinal pigment epithelium–Bruch's membrane complex. Over time, the mechanical stress could predispose to LC or promote ischemia that stimulates angiogenic pathways, leading to mCNV.^6^^,^^7^^,^^10^^,^^11^ Supporting this concept of the role of ischemia in mCNV pathophysiology, OCT angiography (OCTA) studies have shown that choriocapillaris (CC) perfusion is globally reduced in high myopia, particularly in eyes that develop pathologic complications.^12^^–^^14^ However, these prior studies evaluated CC flow over a large macular or peripapillary region without specifically focusing on PSV locations. No study to date has directly assessed whether CC perfusion at PSV sites is locally impaired compared to CC perfusion elsewhere.

In this context, the study was designed with two primary objectives to clarify the potential role of PSV–CC interactions in myopic eyes without features of pathologic myopia: (1) to localize and quantify CC flow deficits at the sites of PSVs using a combined en-face and cross-sectional swept-source OCT/OCTA approach and (2) to identify clinical and anatomic predictors of localized CC impairment at PSV sites, with particular attention to the relevance of age, AXL, and global CC perfusion.

## Methods

### Study Design and Participants

This cross-sectional study included consecutive healthy myopic eyes that were imaged at the Doheny-UCLA Eye Center (Pasadena, CA, USA), Ospedale Luigi Sacco (Milan, Italy), and Città della Salute e della Scienza Hospital (Turin, Italy) between predefined April 2025 and June 2025 window. The study was conducted in compliance with the Health Insurance Portability and Accountability Act and Italian bioethics regulations. The study adhered to the tenets of the Declaration of Helsinki, and informed consent was obtained from each participant.

Inclusion criteria included: refractive error ≤ −0.50 D, clear ocular media, and good quality (signal strength index ≥ 7) swept-source OCTA (SS-OCTA) scans. Exclusion criteria included: history or clinical evidence of posterior staphyloma, subtle structural irregularities (e.g., retinal pigment epithelium undulations), LC, CNV, patchy atrophy, other retinal vascular disease, or systemic conditions affecting ocular circulation, history of ocular surgery or laser photocoagulation, or history of amblyopia, poor scan quality. Both eyes were included in the analysis if they fulfilled eligibility criteria.

### Study Population and Procedures

All subjects were required to have undergone a comprehensive ophthalmic evaluation including detailed medical history collection, best-corrected visual acuity assessment, refraction and spherical equivalent (SE) measurement, intraocular pressure measurement, slit-lamp biomicroscopy, AXL measurement (IOL Master 700, Carl Zeiss Meditec, Dublin, CA, USA), and 6 × 6 mm SS-OCTA scans centered on the fovea (PLEX Elite 9000; Carl Zeiss Meditec). The PLEX Elite system uses a tunable light source laser with a central wavelength of 1050 nm and an acquisition speed of 100,000 A-scans per second. SS-OCTA scans simultaneously provide both flow and structural data. Myopia was defined according to the International Myopia Institute quantitative definition and classification for clinical and epidemiological studies.^15^ Briefly, myopia was defined as SE ≤ −0.50 D, and further classified as low myopia when SE is greater than −6.00 D but ≤ −0.50 D, with high myopia being defined as SE ≤ −6.00 D.

### PSV Identification on Structural En-Face OCT

PSVs were identified through a combined en-face and cross-sectional B-scan OCT analysis as previously described.^9^ PSVs were first identified on volumetric cross-sectional B-scans as hyporeflective tubular structures traversing the sclera toward the choroid and entering into it.^7^^,^^10^ A vessel was defined as a true PSV only if the corresponding B-scan confirmed the presence of a continuous hyporeflective lumen penetrating the sclera and extending into the choroid, thereby excluding spurious hyporeflective signals caused by shadowing, noise, or segmentation artifacts.^10^ Candidate PSVs detected in B-scans were then localized on en-face reconstructions which were generated at the choroid–scleral interface (CSI). A 20 µm thick slab extending 10 µm above and 10 µm below the CSI was used to localize the entry point of PSV into the choroid. On these en-face slabs, the PSV entrance into the choroid appeared as a discrete, hyporeflective interruption within the otherwise continuous CSI.^9^ Only those entry sites confirmed on both en-face OCT CSI slabs and cross-sectional B-scans were included in the analysis. Two independent graders (AQ and AF) masked to the clinical and biometric data performed the identification of PSVs on both en-face and B-scan OCT images using the criteria described above. In cases of disagreement, a senior retinal imaging specialist (SS) adjudicated the final decision. PSVs which had large superficial retinal vessels directly overlying them were excluded from this analysis. Although projection artifact removal algorithms can remove such large vessels, it is unclear whether there may be a residual artifact introduced into the image at the CC level which may confound interpretation. Using the horizontal OCT B-scan passing through the foveal center, the subfoveal choroidal thickness (CT) was measured using the instrument software calipers as the vertical distance between Bruch's membrane and the choroidoscleral border at the foveal center.

### Structural Measurements of PSV and Adjustment for AXL-Related Magnification Effect

Quantitative analysis of PSVs on en-face OCT CSI slabs was performed using ImageJ software (National Institutes of Health, Bethesda, MD). In the reference en-face slab, the region of interest (ROI) was defined by manually tracing the border of the hyporeflective structure at the choroidal entry point, and the area (mm^2^) was automatically computed by the software. The number of PSVs was recorded. Given that CC flow deficits are known to exhibit regional variation (even in healthy eyes) with a tendency toward increased flow deficits in the fovea compared to more peripheral regions of the macula, an additional analysis was conducted to account for and mitigate this potential confounding factor by calculating the perforating scleral vessel-to-fovea distance (VFD). To adjust CC flow deficits percent (CCFD%) for the spatial relationship between each PSV and the foveal center, we performed a pixel to millimeter Euclidean distance analysis. The segmented en-face structural OCT slabs at the CSI from both groups were converted into binary masks. The masks were overlaid with a calibrated square grid, dividing the lesion area into contiguous 0.1 mm² units. The Euclidean distance between each of these centroids and the foveal center was then computed to assess the spatial distribution of PSVs relative to the fovea.

Considering X_i_, Y_i_ = coordinates of each grid point and X_0_, Y_0_ = coordinates of the foveal center, the Euclidean distance was calculated as:

$$d_{i}=\sqrt{{\left(X_{i}-X_{0}\right)}^{2}+{\left(Y_{i}-Y_{0}\right)}^{2}}.$$

The mean of all such distances was then computed to obtain the VFD (d).

$$\bar{d}=\frac{1}{N}\sum_{i=1}^{N}d_{i}.$$

To facilitate coordinate-based spatial analysis, VFD values were decomposed into X, Y coordinates. This method allowed for topography-independent quantification of lesion eccentricity relative to the fovea. To account for AXL-related magnification effects, all linear and area measurements were subsequently corrected post hoc using the Bennett–Littmann method with the formula *F = 3.48 × 0.01306 × (AXL – 1.82),* where *F* represents the magnification factor and *AXL* represents axial length.^12^^,^^16^^,^^17^ Corrected measurements were verified by confirming monotonic scaling with axial length and consistency across repeated measurements. CT/AXL was calculated as an index to normalize CT according to globe size.

### OCTA Analysis

**Figure 1.**
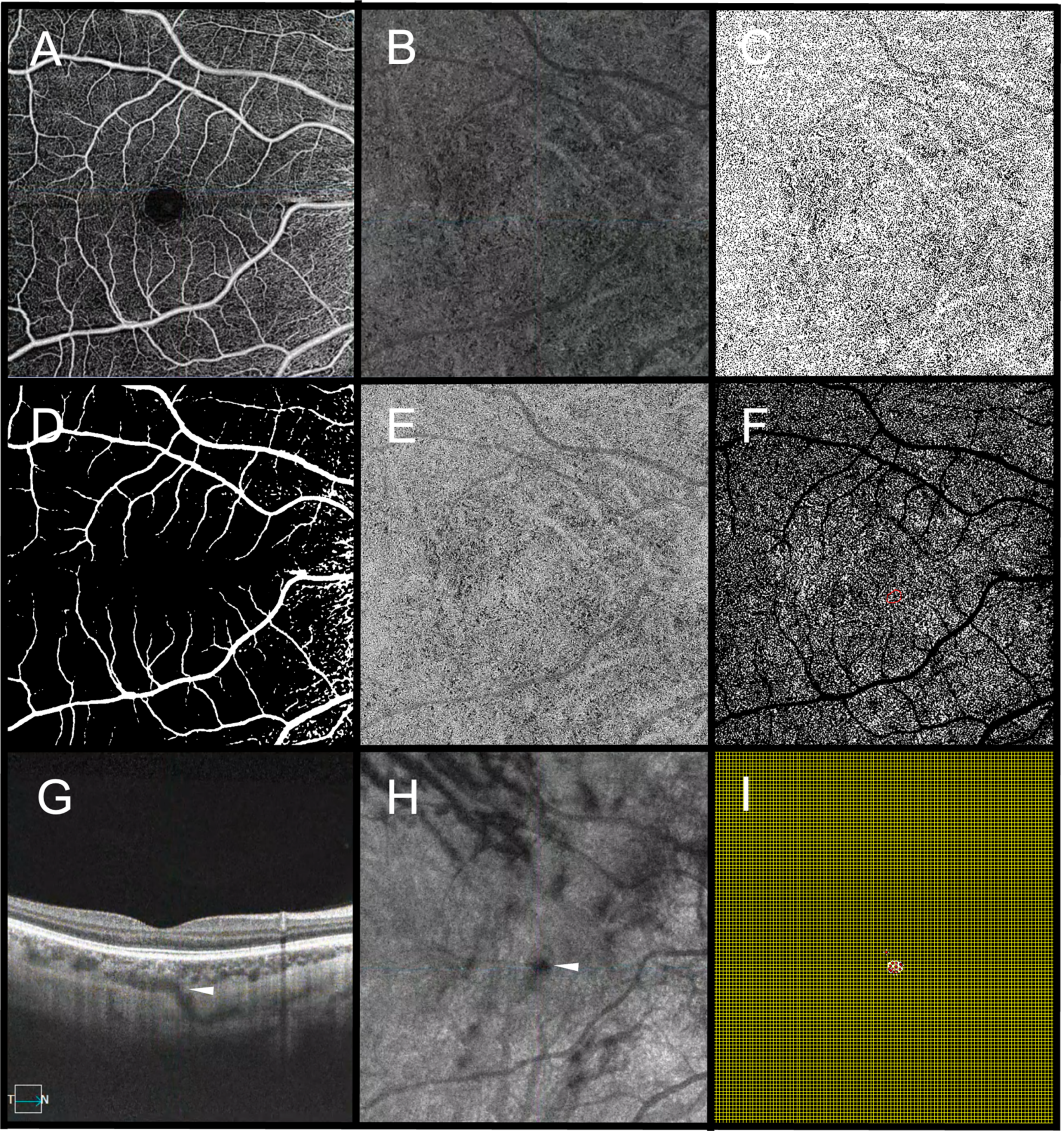
Multimodal image processing workflow for PSV and CC analysis. **(A)** Superficial capillary plexus (SCP) en-face OCTA image showing the retinal vascular network, used for localization and as a reference for masking superficial and large vessels. **(B)** Raw CC en-face OCTA slab, segmented as a 16-µm layer, localized 4 µm beneath the Bruch's membrane, showing flow signal before compensation. **(C)** Compensated and normalized CC en-face OCTA slab, obtained by pixel-wise multiplication of the raw CC en-face OCTA image with the corresponding inverse of the CC en-face OCT slab to correct for potential signal attenuation. **(D)** SCP binarized mask applied to exclude regions beneath large retinal vessels. **(E)** Binarized CC en-face OCTA slab, created from the compensated and normalized CC en-face OCTA image using Phansalkar's local thresholding to delineate flow deficits. **(F)** Final CC en-face OCTA slab with the overlapped SCP binarized mask, used to quantify the percentage of the flow deficits. The *red overlay* highlights the localized PSV. **(G)** Cross-sectional B-scan OCT at the site of PSV entry, confirming a hyporeflective lumen traversing the sclera and entering the choroid. **(H)** En-face generated from the OCT volume showing the circular hyporeflective outline of the perforating vessel (PV) at the choroid entry site (*arrowhead*). **(I)** VFD mapping, with the foveal center manually identified on B-scans. A calibrated grid overlay was applied, and the Euclidean distance from the PSV centroid to the foveal center was computed.

The en-face OCTA CC slab was segmented as a 16-µm thick layer beginning 4 µm below BM.^18^^–^^20^ OCTA slabs were batch processed using a previously validated custom-developed MATLAB (R2022b; MathWorks, Natick, MA, USA) algorithm for signal compensation, and quantitative analysis of CCFDs was performed using ImageJ software (National Institutes of Health, Bethesda, MD, USA).^16^^,^^18^ Briefly, to minimize attenuation artifacts inherent to imaging, the en-face OCTA was compensated by pixel-wise multiplication with the inverse of the corresponding en-face OCT CC image, extracted from the same slab segmentation. The compensated images were subsequently binarized using the Phansalkar local thresholding algorithm with a 23.4-µm radius.^18^^,^^21^ Regions of the CC underlying large retinal vessels were masked and excluded to reduce the influence of shadowing and projection artifacts. It was for this reason that PSVs under major retinal vessels were not included in this analysis. Global CCFD% was quantified as the proportion of the scan area lacking a detectable flow signal, represented as dark pixels in the binary mask.^18^ CCFD% was computed within the corresponding PSV ROI (termed FDPSV) and within a concentric 250-µm-wide ring circumferential to the PSV (FD250) generated from a manually segmented PSV boundary on the same en-face slab (Fig. 1).

### Main Outcome Measures

The primary outcome measure and dependent variables for subsequent analyses were the FDPSV and FD250. These metrics were correlated with clinical, anatomic, and biometric parameters. Candidate predictors were selected a priori based on biological plausibility and prior literature, and included age (years), AXL, SE, CT, global CCFD%, PSV area (mm^2^), and VFD. Each quantitative variable was assessed by two graders, and the mean between the two measurements was used for subsequent analysis.

### Statistical Analysis

Demographic and imaging-derived data were merged at the patient and eye level (patient_ID, eye_ID). Continuous variables were summarized as mean ± SD or median [interquartile range] depending on the distribution as assessed by the Shapiro–Wilk test; categorical variables were expressed as counts and percentages. Associations between predictors and FDPSV were first explored with Spearman rank correlations and univariable mixed-effects models including random intercepts for patient_ID and eye_ID. Multivariable analysis used linear mixed-effects regression with the same random-effects, incorporating all candidate predictors simultaneously. To account for the non-independence of eyes from the same participant, all regression analyses were performed using linear mixed-effects models incorporating nested random intercepts for subject ID and eye ID. This structure explicitly models intra-subject and intra-eye correlation preventing inflation of type I error.^22^ R² was computed for model performance. Models were estimated using maximum likelihood to allow likelihood ratio testing. Model assumptions were verified by examining residual plots (linearity, homoscedasticity, and normality), Q–Q plots, and influence diagnostics (Cook's distance, leverage). (Supplementary Material S1, S2). Multicollinearity was assessed using variance inflation factors, with all values <3.5. Statistical significance was defined as *P* < 0.05. A post-hoc power analysis was performed for the AXL–FDPSV association using the observed regression coefficient and its standard error derived from the 95% CI, estimating power from the noncentral *t*-distribution at *α* = 0.05 and using the subject-level effective sample size according to established regression power references.^23^^,^^24^ Intra-grader repeatability was evaluated in 20 randomly selected eyes, with the grader repeating the segmentation and measurement of PSV area and FDPSV after a two-week interval. Repeatability was quantified using the intraclass correlation coefficient with 95% CI. Analyses were conducted in R version 4.1.0 (R Foundation for Statistical Computing, Vienna, Austria).

## Results

### Cohort Characteristics

Of the initially screened 100 eyes, a total of 52 eyes from 31 healthy myopic participants were included in the final analysis; 48 were excluded for the following reasons: presence of segmentation errors or poor image quality (*n* = 28), incomplete OCT volume or motion artifacts precluding sure PSV visualization at the CSI (*n* = 20). Mean age was 28.30 ± 6.99 years. Mean AXL was 25.28 ± 1.09 mm (range 23.21–27.65 mm), and mean SE was −4.03 ± 1.71 D (range −8.25 to −1.00 D). The cohort was predominantly female (69%). Each eye demonstrated between 1 and 10 PSV, for a total of 119 PSVs (mean 2.3 ± 1.9 per eye) included in the analysis. Mean PSV area was 0.05 ± 0.09 mm^2^ and the mean VFD was 1.50 ± 0.69 mm. Global CCFD% was 17.86% ± 10.58% and FD250 was 18.86% ± 12.13%, whereas the FDPSV was 16.46% ± 12.31%. The mean CT/AXL ratio was 8.34 ± 2.39 (µm/mm). Table 1 shows the complete descriptive statistics for the cohort. Intraclass correlation coefficient was good for all metrics (>0.85).

**Table 1.** Descriptive Characteristics of the Study Population

Variable	Value (Mean ± SD)
Age (years)	28.33 ± 6.99
AXL (mm)	25.28 ± 1.09
AXL distribution	
<26.0 mm	37 eyes
26.0–26.9 mm	10 eyes
≥27.0 mm	5 eyes
SE (D)	−4.03 ± 1.71
CT (µm)	187.01 ± 59.71
PV Counts	2.31 ± 1.94
Gender distribution	
Male	16 eyes (30.8%)
Female	36 eyes (69.2%)
VFD (mm)	1.50 ± 0.69
Horizontal PV Position (XPV)	0.09 ± 1.11
Vertical PV Position (YPV)	0.61 ± 1.09
PSV Area (mm²)	0.052 ± 0.09
Global CCFD (%)	17.86 ± 10.58
FD250 (%)	18.86 ± 12.13
FDPSV (%)	16.46 ± 12.31

AXL, Axial Length; CT, Choroidal Thickness;CCFD, Choriocapillaris Flow Deficit; FD250, Flow Deficit at 250 µm ring from Perforating Scleral Vessel site; PSV, Perforating Scleral Vessel; SE, Spherical Equivalent; VFD, Vessel to Fovea Distance.

Summary statistics of demographic, ocular biometric, and imaging-derived parameters for the included 52 eyes from 31 healthy myopic participants. Continuous variables are expressed as mean ± SD, and categorical variables as counts and percentages.

### Correlations Analyses

**Figure 2.**
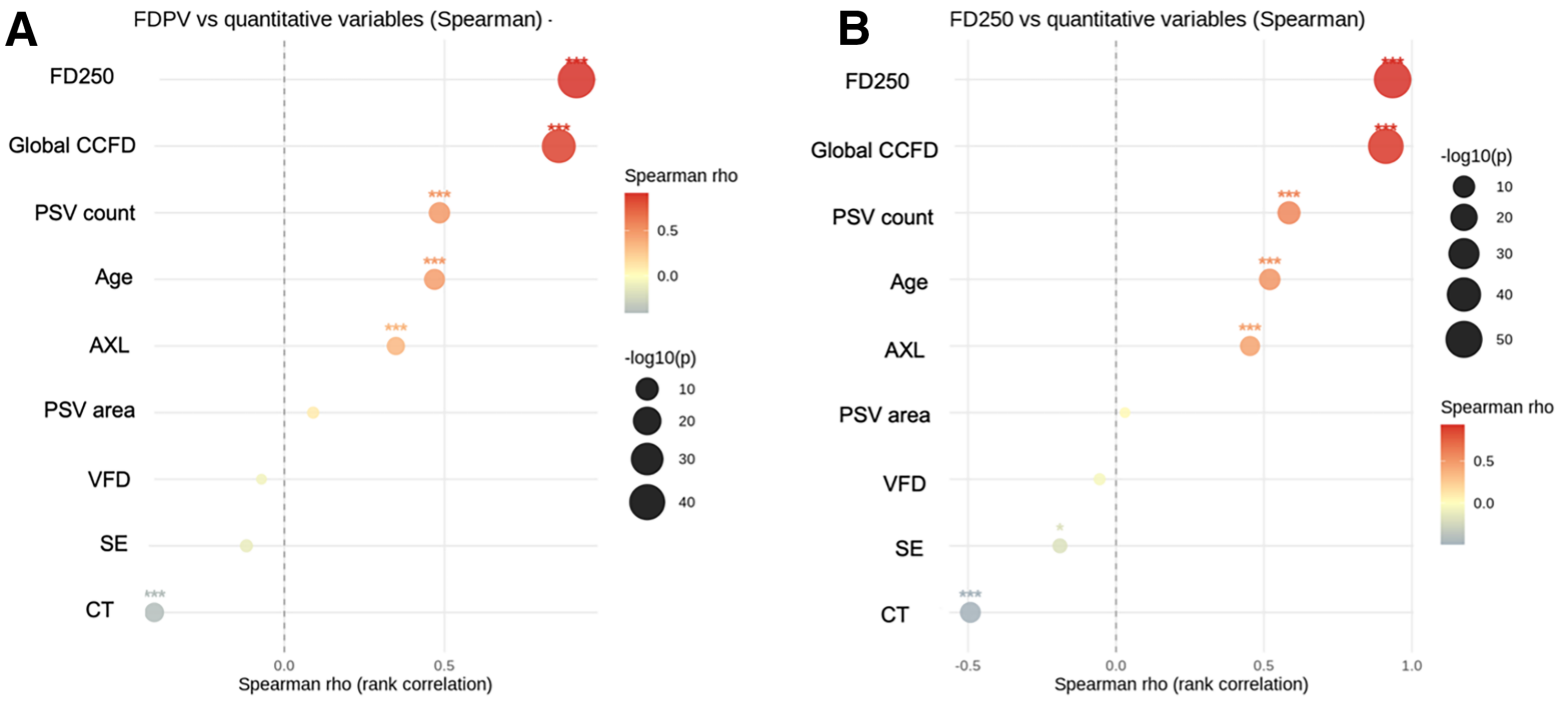
Spearman correlation analysis between demographic, biometric, and structural variables and choriocapillaris flow deficits. Bubble plots illustrate the strength and significance of correlations between candidate predictors and (**A**) flow deficits at PSV sites (FDPV), and (**B**) flow deficits within a 250-µm perivessel window (FD250). The x-axis represents Spearman's rho, with positive correlations shown to the right of the vertical dashed line and negative correlations to the left. Bubble color indicates correlation strength (*red* = stronger positive, *blue* = stronger negative), whereas bubble size encodes significance level (−log10 *P* value). Significant correlations are denoted with *asterisks* (*P* < 0.05, *P < 0.01, **P < 0.001).

**Figure 3.**
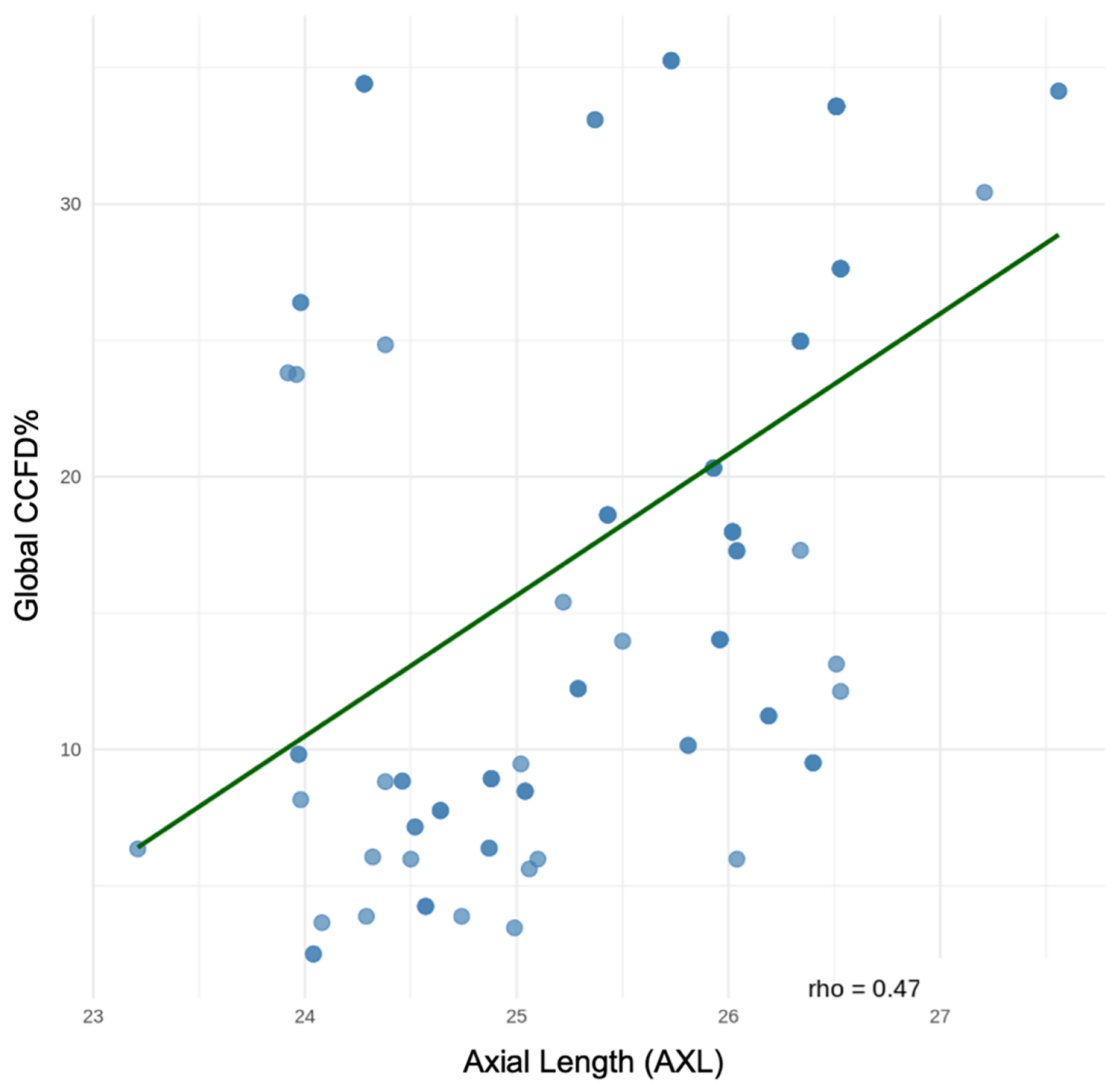
Scatterplot showing the relationship between AXL and global choriocapillaris flow deficits (CCFD%). A significant positive correlation was observed (Spearman's rho = 0.47, *P* < 0.001), indicating that longer axial length is associated with greater global CCFD. The fitted regression line (*green*) illustrates the increasing trend of CCFD% across the observed AXL range.

Spearman correlation analyses demonstrated that both FDPSV and FD250 were significantly positively associated with age, AXL, and global CCFD% (all *P* < 0.001). PSV count also correlated positively with FDPSV and FD250 (*P* < 0.001). In contrast, the PSV area and VFD did not show consistent associations (*P* > 0.05) (Fig. 2). Global CCFD% positively correlated with AXL (*ρ* = 0.47, *P* < 0.001) (Fig. 3).

### Univariable Analyses

In a univariable mixed-effects model, older age (*β* = 0.96; 95% CI, 0.49–1.43; *P* < 0.001), AXL (*β* = 2.03; 95% CI, −1.67 to 5.65; *P* = 0.273), and global CCFD (*β* = 0.99; 95% CI, 0.87–1.11; *P* < 0.0001) were positively associated with FDPSV. PSV area demonstrated a moderate association without reaching statistical significance (*β* = 13.38; 95% CI, −0.45 to 27.25; *P* = 0.059). SE, CT, and VFD did not significantly predict FDPSV, as shown in Table 2.

**Table 2.** Univariable Mixed-Effects Model for Predictors of FDPSV

Predictors	Estimate	95% CI	*P* Value
Age (years)	0.963	0.492 to 1.426	<0.001
AXL (mm)	2.027	−1.671 to 5.649	0.273
SE (D)	0.079	−1.857 to 2.050	0.936
CT (µm)	−0.001	−0.053 to 0.052	0.972
Global CCFD (%)	0.989	0.874 to 1.111	<0.0001
PSV Area (mm^2^)	13.380	−0.454 to 27.251	0.059
VFD (mm)	−0.850	−2.937 to 1.248	0.423

FDPV, flow deficits at perforating vessel site; PV, perforating vessel.

The table summarizes the results of univariable mixed-effects analyses evaluating the association between demographic and structural covariates and FDPSV. Each model included a single fixed effect predictor with patient_ID and eye_ID entered as random intercepts to account for inter-subject and inter-eye correlations.

### Multivariable Analyses

In the final multivariable mixed-effects model, age (*β* = 0.23; 95% CI, 0.01–0.45; *P* = 0.045) and global CCFD% (*β* = 1.02; 95% CI, 0.86–1.19; *P* < 0.0001) were still independent positive predictors of FDPSV. Interestingly, AXL emerged as a negative predictor (*β* = −2.37; 95% CI, –4.14 to −0.60; *P* = 0.010), indicating reduced flow deficits at PSV sites in longer eyes despite worsening global CCFD. PSV area was also significant (*β* = 16.52; 95% CI, 4.37–28.66; *P* = 0.009), whereas SE, CT, and VFD were not associated, as shown in Table 3. *R*² was 0.24.

**Table 3.** Fixed-Effects Estimates From the Multivariable Mixed-Effects Model Predicting FDPSV

Predictors	Estimate	95% CI	*P* Value
Intercept	48.529	5.837 to 91.222	0.028
Age (years)	0.230	0.007 to 0.451	0.045
AXL (mm)	−2.372	−4.141 to −0.604	0.010
SE (D)	−0.434	−1.514 to 0.647	0.433
CT (µm)	0.013	−0.010 to 0.036	0.281
Global CCFD (%)	1.022	0.857 to 1.187	<0.001
PSV Area (mm^2^)	16.518	4.373 to 28.662	0.009
VFD (mm)	−1.153	−2.828 to 0.523	0.180

The table presents the results of the multivariable model including age, AXL, SE, CT, global CCFD, PV area, and VFD as predictors of FDPSV with patient_ID and eye_ID entered as random intercepts to account for inter-subject and inter-eye correlations.

## Discussion

In this study of 52 eyes from 31 otherwise healthy myopic adults, we investigated how PSVs, which are presumed short posterior ciliary arteries, are associated with CC perfusion within a non-pathologic context. We quantified CC flow deficits at corresponding PSV entry points into the choroid (FDPSV) and within a 250-µm perivessel zone (FD250). Univariable analyses confirmed that older age and worse global CCFD were significantly associated with greater FDPSV. However, multivariable modeling revealed a surprising negative association between AXL and FDPSV after accounting for age, global CCFD, and PSV area. Although axial elongation typically exacerbates global CC ischemia, our findings of reduced FD at PSV sites with increasing AXL suggest anatomical remodeling at the PSV zone and notably, the CCFD% immediately surrounding the PSV was relatively increased. Regardless, this divergence underscores the importance of considering both global and focal perfusion mechanisms when interpreting CC changes in myopia.

Healthy myopic eyes without pathological fundus findings offer a unique opportunity to investigate the earliest structural–vascular interactions between PSVs and the CC in the absence of degenerative changes, LC, or mCNV. The demographic profile of our subjects positions our study cohort in the spectrum of early/non-pathologic myopia. Our cohort (younger, non-pathologic myopes) contrasts with the predominantly older, highly myopic/pathologic cohorts in which PSVs have been implicated to be of pathophysiologic relevance.^7^^,^^10^^,^^25^^–^^27^ This framing is important because the International Myopia Institute emphasizes that high myopia (refractive magnitude) and pathologic myopia (specific lesions) are not interchangeable; early myopia may therefore exhibit a different spectrum of microvascular alterations than more advanced high myopia or pathologic myopia stages.^1^^,^^15^^,^^16^^,^^28^ Studying these earlier or non-pathologic stages may provide insights into the evolution and pathophysiology of changes observed in the later stages.

Consistent with previous studies by Su et al.^12^ and Al-Sheikh et al.,^13^ we confirmed a substantial degree of global CCFD% in our cohort, highlighting the progressive CC rarefaction that has been described with increasing axial elongation. These global trends provide a context for our local PSV-site analysis. The PSV global CCFD% difference was small relative to variability, with potential attenuation and influence by unmeasured local and global factors. The deficits were strongly correlated with age and AXL, suggesting that PSV sites also reflect the broader CC rarefaction observed throughout the posterior pole with age. Notably, PSV area and VFD did not show independent effects in univariable testing, suggesting that an individual vessel's entry size or macular eccentricity is less influential than AXL or age at this early stage.^28^ This reinforces the established relationship between axial elongation, aging, and CC attenuation in myopic eyes, consistent with histopathologic analyses by Hirata and Negi^29^ and in vivo topographical studies.^16^^,^^30^^,^^31^ While PSV presence may be important, their absolute size or proximity to the fovea may exert less influence on CC ischemia than other determinants of choroidal health.

Notably, the focus on PSV areas did not reveal substantially larger deficits directly over the ROI compared to immediately adjacent regions (FD250) implying that CC at PSV sites does not exhibit isolated or highly localized ischemia in early myopic eyes, and in fact, the opposite was observed. Population-based analyses outside myopia also emphasize that CCFD% scales with ocular/biologic determinants, reinforcing our finding that global CCFD% strongly predicts local PSV-site deficits.^13^

Pairwise correlations between AXL, FDPSV, and CCFD% were all positive and consistent with prior work.^12^^,^^13^ Age and global CCFD% were still strong positive predictors of FDPSV, consistent with cumulative localized CC compromise over time. However, AXL demonstrated an inverse association with FDPSV after adjusting for covariates. The shift from a positive univariable to a negative multivariable association between AXL and FDPSV reflects the potential influence of the other biologically relevant covariates in the model, which modify the adjusted relationship through shared variance. This counterintuitive finding suggests that in early-stage myopia, axial elongation may induce scleral remodeling or posterior contour changes that alter PSV orientation, thereby reducing direct mechanical compression of the overlying CC by the PSV. Although axial elongation is typically associated with worsening global CC ischemia, the observation that FDPSV decreases with increasing AXL suggests that these vessels may confer localized preservation of perfusion. This anatomical adaptation may mitigate focal ischemia even as overall perfusion worsens. Because all linear and areal measurements were uniformly corrected using the Bennett–Littmann method and the corrected ROI geometry was verified for consistency, the observed inverse association between AXL and FDPSV cannot be attributed to magnification artifacts and it likely reflects true anatomical or physiological variation rather than a scaling issue. Support for this interpretation comes from MRI-based studies by Moriyama et al.,^32^^,^^33^ which document irregular posterior curvature and scleral thinning that may suggest possible repositioning of PSV trajectories. Additional studies supported that connective-tissue remodeling of the sclera influences choroidoscleral biomechanical changes in myopia likely impacting on structural shifts in the PSV–CC interface.^34^^,^^35^ Although no eyes in this cohort demonstrated staphyloma, subtle posterior pole contour irregularities may still have influenced our findings.^32^ Another possibility is that PSVs act as relative “oases” of blood supply, maintaining CC integrity in their vicinity despite global hypoperfusion, consistent with histopathological evidence that CC lobules are organized around feeder sites.^36^^–^^39^ A physiological compensatory role is also plausible, whereby PSVs increase their functional contribution to perfusion demands in elongated eyes, thus buffering localized regions of the CC.^36^^,^^38^ Elongated eyes may alter the incidence of the OCT scanning beam relative to the segmentation planes leading to artifactual “reduction” of local CC flow deficits at PSV site, a concept supported by the previous work of Dolz-Marco and Freund in which scan tilting (such as in staphylomatous eyes) in peripheral zones was found to influence OCTA assessment from the deep capillary plexus and beyond.^40^ Taken together, the negative association between AXL and CCFD% at PSV sites likely reflects an interplay of anatomical remodeling, localized vascular preservation, and possible methodological factors, underscoring the need to distinguish global from focal perfusion dynamics when evaluating choroidal health in myopia. Given the absence of direct measurements of posterior curvature or scleral geometry, the proposed explanation for the inverse AXL–FDPSV association should be regarded as an exploratory hypothesis rather than an established biological mechanism. Further studies should investigate how these factors could influence this association.

Although prior studies have emphasized the relevance of PSVs in the pathophysiology of complications of pathologic myopia, our study highlights their role in shaping local CC perfusion in healthy myopes. Our results suggest that even in eyes that are myopic but not pathologic the entry of PSV into the choroid may create localized alterations in the CC perfusion. Because CC ischemia is thought to be a driver of the development of subsequent mCNV in myopes, the apparent relatively better perfusion at PSV sites might suggest that MNV may be less likely to develop these locations. This hypothesis, however, will need to be evaluated in future longitudinal studies, as our cross-sectional design unfortunately limits causal inferences. We also did not directly assess posterior curvature or scleral topography, limiting the ability to statistically test the proposed geometric explanations. Future research should integrate wide-field OCTA with posterior pole shape modeling and ideally enroll eyes across the myopia continuum from emmetropia through high myopia and pathologic myopia stages to clarify how PSV–CC interactions evolve. Longitudinal and multimodal imaging studies are warranted to establish PSV-site behavior across myopia progression and its relevance to disease progression or prevention. Yet, paradoxically, in elongated but otherwise non-pathologic myopic eyes, PSV-related deficits may attenuate, possibly reflecting adaptive remodeling. Because the study cohort consisted primarily of young adults with low-to-moderate non-pathologic myopia, the generalizability of our findings is limited to earlier stages of myopic remodeling. Structural–vascular interactions at PSV sites may differ substantially in high or pathologic myopia, and future studies including a broader myopia spectrum are required to determine whether the observed relationships persist in more advanced disease*.* This apparently nonlinear influence of PSVs across the myopia spectrum ideally requires longitudinal studies incorporating ultra-widefield OCTA and enhanced depth imaging. Several methodological limitations should be acknowledged, including the non-random exclusion of PSVs located beneath large retinal vessels, the use of a single OCTA platform with proprietary processing algorithms that may limit reproducibility on other systems, and the possibility that unmeasured scan tilt or segmentation-plane angle variability could influence CC attenuation patterns. Moreover, since FDPSV data were not available for age-matched non-myopic controls, direct comparison with emmetropic eyes could not be performed. Future studies should assess the impact of these factors on the reported measurements.

## Conclusions

Our results suggest that the entry sites of PSVs may serve as points of relative CC support, with their functional impact being modulated by stage-specific structural dynamics. The percentage of CC flow deficits at PSV sites was consistently lower than those observed in their immediate perivascular surroundings and the global CC, suggesting that PSVs may confer localized preservation of CC perfusion. These findings may reflect an early adaptive increase in localized CC perfusion around PSVs in non-pathologic myopia, a response that may become insufficient once axial elongation surpasses a threshold ultimately contributing to the degenerative changes observed in high myopia. Further longitudinal studies will be essential to determine whether the relative preservation of PSV-associated CC flow represents a protective factor or, conversely, a precursor to localized stress and subsequent chorioretinal degeneration.

## Supplementary Material

Supplement 1
