# Supplementary figures and images for: Localized Choriocapillaris Flow Deficits at Perforating Vessel Sites in Myopic Eyes
Source: Invest Ophthalmol Vis Sci. 2026 Jan 20;67(1):43. doi: 10.1167/iovs.67.1.43 (PMC12831146; doi:10.1167/iovs.67.1.43)

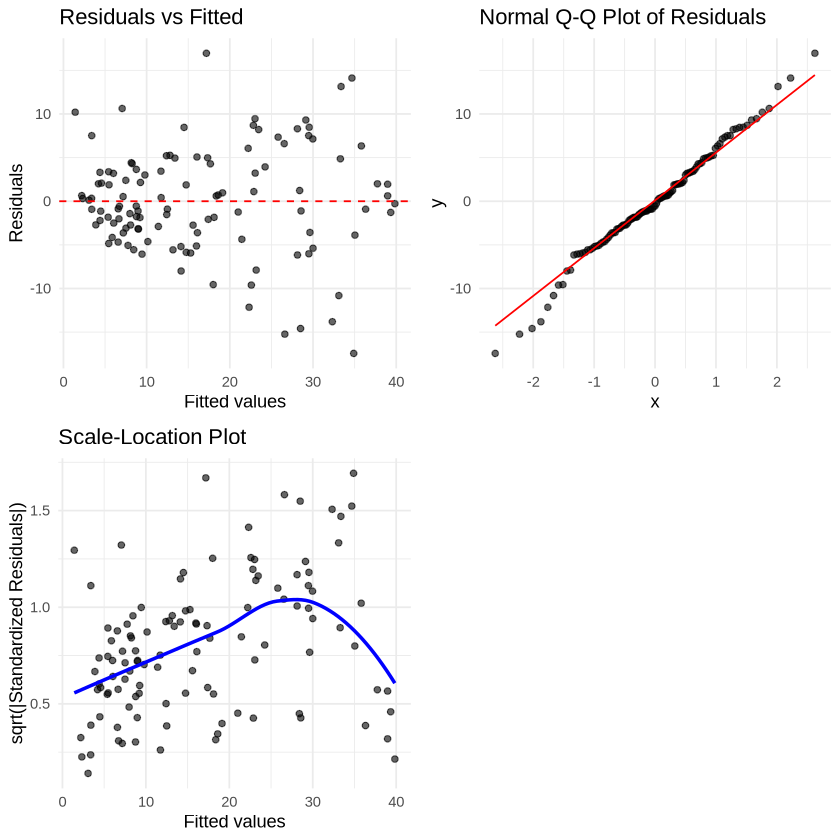


Supplementary Material 1


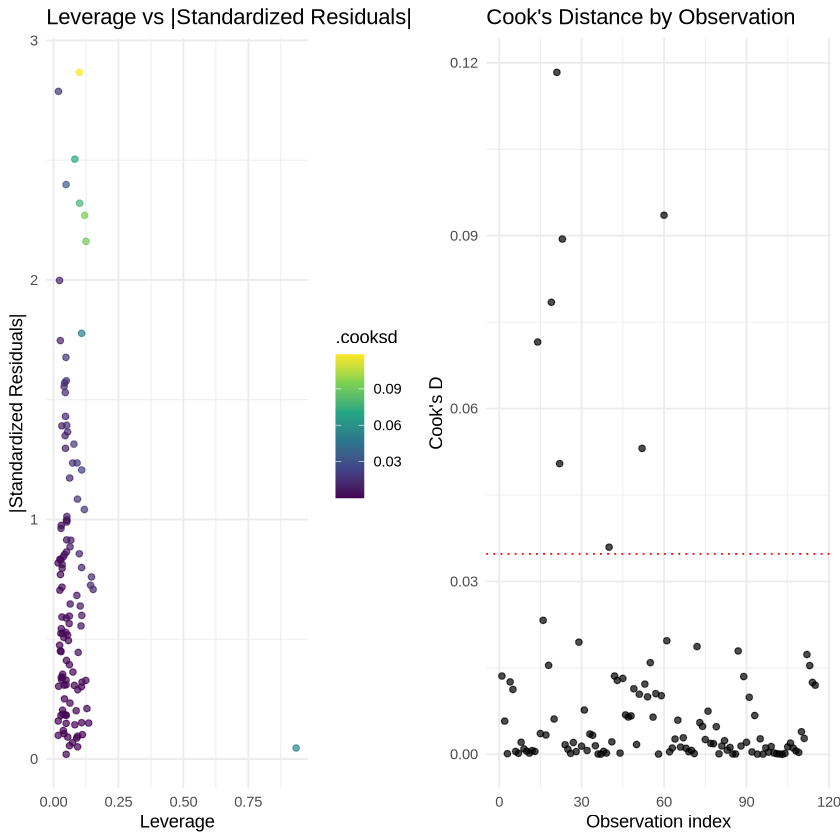


Supplementary Material 2

Supplement: Supplement 1 [file iovs-67-1-43_s001.docx]
